# Prediction and validation of murine MHC class I epitopes of the recombinant virus VSV-GP

**DOI:** 10.3389/fimmu.2022.1100730

**Published:** 2023-01-19

**Authors:** Saskia V. Vijver, Sarah Danklmaier, Lisa Pipperger, Raphael Gronauer, Gabriel Floriani, Hubert Hackl, Krishna Das, Guido Wollmann

**Affiliations:** ^1^ Institute of Virology, Medical University of Innsbruck, Innsbruck, Austria; ^2^ Christian Doppler Laboratory for Viral Immunotherapy of Cancer, Medical University of Innsbruck, Innsbruck, Austria; ^3^ Institute of Bioinformatics, Medical University of Innsbruck, Innsbruck, Austria; ^4^ ViraTherapeutics GmbH, Innsbruck, Austria

**Keywords:** oncolytic virus, epitope, prediction, VSV (vesicular stomatitis virus), MHC-I, T cell

## Abstract

Oncolytic viruses are currently tested as a novel platform for cancer therapy. These viruses preferentially replicate in and kill malignant cells. Due to their microbial origin, treatment with oncolytic viruses naturally results in anti-viral responses and general immune activation. Consequently, the oncolytic virus treatment also induces anti-viral T cells. Since these can constitute the dominant activated T cell pool, monitoring of the anti-viral T cell response may aid in better understanding of the immune responses post oncolytic virotherapy. This study aimed to identify the anti-viral T cells raised by VSV-GP virotherapy in C57BL/6J mice, one of the most widely used models for preclinical studies. VSV-GP is a novel oncolytic agent that recently entered a clinical phase I study. To identify the VSV-GP epitopes to which mouse anti-viral T cells react, we used a multilevel adapted bioinformatics viral epitope prediction approach based on the tools netMHCpan, MHCflurry and netMHCstabPan, which are commonly used in neoepitope identification. Predicted viral epitopes were ranked based on consensus binding strength categories, predicted stability, and dissimilarity to the mouse proteome. The top ranked epitopes were selected and included in the peptide candidate matrix in order to use a matrix deconvolution approach. Using ELISpot, we showed which viral epitopes presented on C57BL/6J mouse MHC-I alleles H2-Db and H2-Kb trigger IFN-γ secretion due to T cell activation. Furthermore, we validated these findings using an intracellular cytokine staining. Collectively, identification of the VSV-GP T cell epitopes enables monitoring of the full range of anti-viral T cell responses upon VSV-GP virotherapy in future studies with preclinical mouse models to more comprehensively delineate anti-viral from anti-tumor T cell responses. These findings also support the development of novel VSV-GP variants expressing immunomodulatory transgenes and can improve the assessment of anti-viral immunity in preclinical models.

## Graphical Abstract

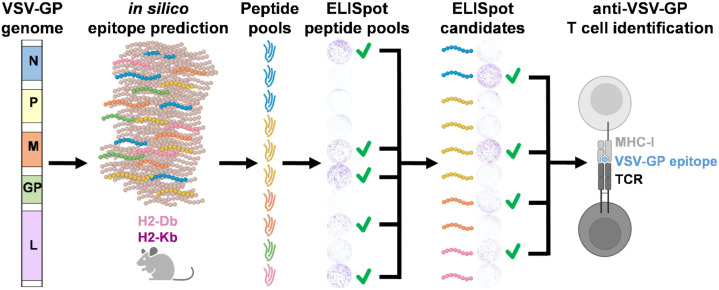

## 1 Introduction

Oncolytic virotherapy is a relatively new cancer treatment modality which makes use of oncolytic viruses (OVs) that preferentially infect and kill malignant cells (1). The oncolytic virus VSV-GP is a chimeric, replication-competent, enveloped, negative-strand RNA virus based on the vesicular stomatitis virus (VSV) carrying the lymphocytic choriomeningitis virus glycoprotein (LCMV-GP) in place of the VSV glycoprotein G (2). Advantages of VSV-GP for oncolytic virotherapy are the fast replication cycle, the lack of pre-existing immunity in humans and the reduced induction of neutralizing antibodies (3). Oncolytic virotherapy with VSV-GP has shown promise in preclinical mouse models of glioblastoma (2), ovarian carcinoma (4), melanoma (5), prostate (6), and lung cancer (7). VSV-GP can also carry additional transgenes such as tumor antigens (8) or diverse therapeutic modulators as has been shown with other VSV variants (9).

However, in oncolytic virotherapy, not only anti-tumor T cells but also anti-viral T cells are present in the tumor, since virus treatment induces anti-viral T cells as well, which can constitute the dominant activated T cell pool (10). This anti-viral immunity receives much less attention in preclinical, lest to mention, clinical studies although it may either interfere with anti-tumor T cells (8) or contribute to anti-tumor immunity by multiple indirect mechanisms (11). As we have previously reported, VSV-GP can induce T cell homing to the tumor (7), thereby turning the cold tumor hot, similarly to other OVs (12). This can potentially enhance preexisting anti-tumor immunity, and offers opportunities for combination of OVs, such as VSV-GP, with other therapeutic modalities, such as checkpoint inhibitors (11).

In order to differentiate between anti-tumor and anti-viral T cells in the tumor, anti-viral T cells against VSV-GP epitopes need to be identified. These epitopes are short peptides originating from antigen processing of the viral proteins and subsequently, epitopes are presented by the major histocompatibility complex (MHC) to enable T cell recognition (13). The peptide binding grooves of different MHC alleles vary, thereby resulting in differential epitope presentation (13). Since the T cell receptor (TCR) recognizes the MHC-peptide complex, not only the epitope itself but also the MHC allele affects TCR recognition (14). The antigen processing and presentation can be predicted based on databases of known presented epitopes (15), which are a powerful tool to predict epitope presentation, and can be used for viral epitope prediction as well. Widely used mouse models for oncolytic virus research also present multiple MHC-I alleles, such as H2-Db and H2-Kb for C57BL/6J mice (16).

Therefore, this study aimed to identify the anti-viral CD8^+^ T cells against the recombinant VSV-GP in the C57BL/6J mouse model, using multilevel bioinformatic viral epitope prediction, ELISpot and flow cytometry. Investigating the anti-VSV-GP T cells could improve the preclinical development of novel VSV-GP variants by advancing assessment of the anti-viral immunity in mouse models, may contribute to monitoring of vector immunity raised by VSV-based vaccines, and aid in further development of VSV-based vaccines.

## 2 Materials and methods

### 2.1 Epitope predictions

The genome sequence of VSV-GP was used (2). Epitope predictions were performed using the corresponding amino acid (AA) sequences of the VSV-GP proteins. Three prediction tools were applied to predict MHC-I binding of 8-11mer peptides to H2-Db and H2-Kb and peptide immunogenicity: netMHCpan 4.1 with eluted ligands and binding affinity training data (17), MHCflurry 2.0 (18), and netMHCstabPan (19). Percentile rank scores were estimated from predicted binding values from a set of random background sequences for the respective MHC alleles. Peptides were defined as ‘strong binders’ when their percentile rank scores were smaller than 0.5%, ‘weak binders’ when their percentile rank scores were 0.5-2%, and ‘marginal binders’ when their percentile rank scores were 2-3%. All predicted epitopes from the three bioinformatic tools were ranked based on their consensus of the binding strength (strong, weak and marginal binders), rank score for predicted stability for the peptide-MHC interaction, and dissimilarity to the mouse proteome (GRCm38 Ensembl release 90) using the antigen.garnish package (20). Additionally, the percentage of hydrophobic amino acids and the foreignness score were computed but were not used for epitope ranking. Due to viral sequential gene expression of VSV-GP (21), a decreasing number of candidate epitopes of each viral proteins were selected from the top-ranked epitopes. Per MHC-I allele, 15 VSV-N, 15 VSV-P, 10 VSV-M, 5 LCMV-GP and 5 VSV-L candidate epitopes were included in the peptide candidate matrix.

### 2.2 Peptides

The candidate peptides, in total 100 peptides, were purchased from JPT Peptide Technologies (Berlin, Germany). After synthesis, the peptides were analyzed using HPLC and mass spectrometry to assess purity (on average 73% for H2-Db peptides and 77% for H2-Kb peptides). Subsequently the peptides were pooled into 14 peptide pools per MHC-I allele. The lyophilized peptides were reconstituted in DMSO (Sigma-Aldrich/Merck #2650, Taufkirchen, Germany) and stored at -80°C. Directly before addition to the ELISpot plate, the peptide solution was further diluted to indicated concentrations in T cell medium consisting of DMEM high glucose (Sigma-Aldrich #D5671) supplemented with 10% Fetal Bovine Serum (FBS; PAN-Biotech #P30-3306, Aidenbach, Germany), 1% Penicillin/Streptomycin (#15140-122; equivalent to 100 U/ml and 100 μg/ml respectively), 10 mM HEPES (#15630-056), 1X non-essential amino acids (NEAA; #11140-050), 1X GlutaMAX (#A12860-01) and 50 µM β-mercaptoethanol (#31350-010) purchased from Gibco (Carlsbad, CA, USA).

### 2.3 Ethical approval of mouse experiments

Mouse experiments were approved by the Institutional Review Board of the Medical University of Innsbruck (ZVTA) and the Federal Ministry of the Republic of Austria for Education, Science and Research (BMBWF; license 2020-0.475.503).

### 2.4 Mice and housing

Six- to eight-week-old C57BL/6JRj mice were purchased from Janvier Labs (Le Genest St Isle, France). Animals were housed in groups in individually ventilated cages in a BSL2 facility at 20-24°C, approximately 55% humidity, ad libitum feeding and water, and with a day-night cycle of 12 hours (h). No animals were excluded from the described experiments. The exact animal numbers for each experiment are given in the figure legends.

### 2.5 Virus immunization of mice and tissue harvest

VSV-GP was produced as previously described (8) and titrated using a TCID_50_ assay on BHK-21 cells using the Spearman-Kärber method (22). Mice were immunized with 1 x 10^8^ TCID_50_ VSV-GP in PBS (Sigma-Aldrich #D8537) intravenously (i.v.) into the lateral tail vein using a 30G needle.

One week post immunization, mice were anaesthetized with isoflurane and sacrificed using cervical dislocation. Spleens were harvested and processed into single-cell suspension using mechanical dissociation over a 40 µm cell strainer. Erythrocytes were lysed using BD Pharm Lyse (BD #555899, San Jose, CA, USA) and splenocytes were subsequently resuspended in T cell medium.

### 2.6 Enzyme-linked immunospot (ELISpot)

The IFN-γ ELISpot was performed using a mouse IFN-γ ELISpot kit (Mabtech #3321-4APT-10, Cincinnati, OH, USA) following the manufacturer’s protocol. Briefly, ELISpot plates were washed with PBS and blocked with DMEM high glucose containing 10% FBS. Peptide solution in T cell medium was added to the plates and 2.5 x 10^5^ splenocytes were added in T cell medium. The exact peptide concentrations for each experiment are indicated in the figure legends. As positive control, concanavalin A (ConA; Sigma-Aldrich #C0412) in T cell medium was added to a final concentration of 5 µg/ml. Plates were wrapped in aluminum foil and incubated for approximately 18 h at 37°C with 5% CO_2_. The next day, cells were removed and plates were washed with PBS. Plates were incubated with 1 µg/ml detection antibody in PBS supplemented with 0.5% FBS for 2 h at room temperature (RT). Plates were washed with PBS again and were incubated with 1 µg/ml streptavidin-ALP in PBS with 0.5% FBS for 1 h at RT. Subsequently, plates were washed with PBS and were developed with BCIP/NBT-plus substrate. Development was stopped by rinsing the plates under tap water.

ELISpot plates were air-dried overnight and scanned using the ImmunoSpot S6 Ultra V Analyzer (CTL, Shaker Heights, OH, USA). Spots were counted using the ImmunoSpot software version 7.0.20 based on published guidelines (23), and spot counts of technical replicates were averaged.

### 2.7 Intracellular cytokine staining (ICS)

Single-cell suspensions containing 7.5 x 10^5^ splenocytes were plated in 96-well U-bottom plates in T cell medium. Afterward, either peptide solution (10 µg/ml), or as a positive control phorbol-12-myristate-13-acetate (PMA; Sigma-Aldrich #P8139; 10 µg/ml) together with ionomycin (Sigma-Aldrich #I0634; 1 µg/ml) in T cell medium was added. After 5 h at 37°C and 5% CO_2_ GolgiPlug (BD #555029; 1 µl/ml) was added and samples were incubated for an additional 10 h.

Cells were resuspended in FACS buffer consisting of PBS supplemented with 2% FCS and 5 mM EDTA and were stained using LIVE/DEAD Fixable Near-IR Dead Cell Stain (ThermoFisher #L34976, 1:1000 dilution), anti-mouse CD45.2-PerCP-Cy5.5 (Biolegend #109828; 1:200 dilution), anti-mouse CD3-AF488, (Biolegend #100210; 1:200 dilution), anti-mouse CD8a-BV421 (Biolegend #100737; 1:200 dilution) and anti-mouse CD4-BV510 (Biolegend #100449; 1:200 dilution) for 30 minutes (min) at 4°C in the dark. Subsequently, cells were washed twice with FACS buffer and permeabilized and fixed using Cytofix/Cytoperm (BD #554722) for 30 min at RT. Permeabilization/fixation was stopped by washing twice with 1X Perm/Wash buffer (BD #554723). Intracellular staining with anti-mouse IFN-γ-APC (Biolegend #505810; 1:100 dilution), as well as the corresponding isotype control (IgG1-APC, Biolegend #400411), was performed in 1X Perm/Wash buffer for 30 min at 4°C. After washing twice, samples were acquired on a FACS Canto II (BD Biosciences) and analyzed using the FlowJo software (version 10.8.1, BD Biosciences).

### 2.8 Data and statistical analyses

Data visualization and statistics were performed in GraphPad Prism version 9.3.1 (GraphPad Software, La Jolla, CA, USA). Sequence logos of identified epitopes were created using the Weblogo tool (24) where all peptide sequences start at position one of the logo. Unless otherwise specified, points indicate the average of technical replicates, bars represent the means and the error bars indicate the standard deviation. The statistical tests that were used are indicated in each figure legend. A p-value lower than 0.05 was considered statistically significant, and the significant differences are indicated in each graph: * p < 0.05; ** p ≤ 0.01; *** p ≤ 0.001; **** p ≤ 0.0001.

## 3 Results

### 3.1 Workflow and epitope predictions for H2-Db and H2-Kb alleles

**Figure 1:**
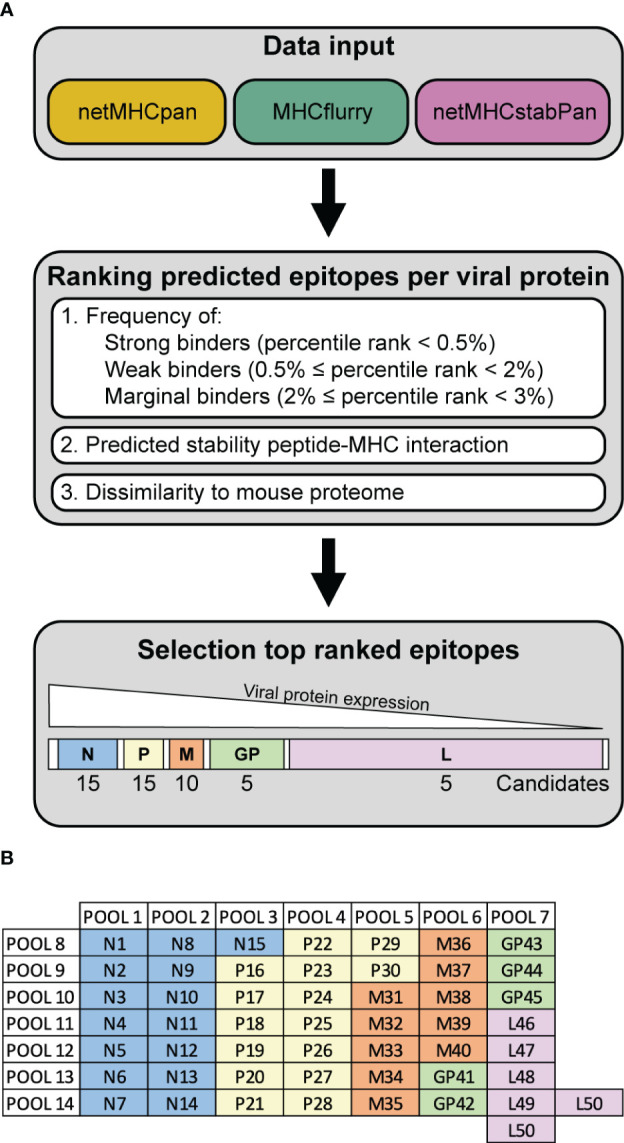
Workflow VSV-GP epitope predictions. **(A)** Schematic representation of the steps of the epitope prediction workflow. **(B)** Exemplary peptide candidate matrix for both H2-Db and H2-Kb. For additional information on epitope predictions, see **Supplementary Figure 1**.

To investigate the anti-viral T cells induced upon recombinant VSV-GP treatment in mouse models, we performed epitope predictions for the five VSV-GP proteins and their presentation by the mouse MHC-I alleles in C57BL/6J mice, H2-Db (in short ‘Db’) and H2-Kb (in short ‘Kb’). We used an integrative multilevel bioinformatic epitope prediction approach based on the tools netMHCpan 4.1 (17), MHCflurry 2.0 (18), and netMHCstabPan (19), which are commonly used in neoepitope identification. The bioinformatics approach including the selection criteria is depicted in **Supplementary Figure 1**. Based on the ranking of the predicted epitopes (**Figure 1A**), 50 predicted epitopes per MHC-I allele were selected and included in the peptide candidate matrix, where each peptide was included in one vertical and in one horizontal pool (**Figure 1B**). A characteristic feature of non-segmented negative strand RNA viruses is the sequential gene expression resulting in a gradient of gene products starting from the 3’ transcription initiation site (21). Consequently, the expression of the viral VSV-GP proteins decreases towards the 5’ end of the VSV-GP genome, meaning VSV-N shows the highest expression and VSV-L shows the lowest expression. Therefore, a decreasing number of candidate epitopes of each viral protein was selected and arranged into a peptide candidate matrix consisting of horizontal and vertical peptide pools (**Supplementary Figure 2**). The highest ranked epitopes per viral protein were selected and therefore 15 VSV-N, 15 VSV-P, 10 VSV-M, 5 LCMV-GP and 5 VSV-L epitopes were arranged into the peptide candidate matrix. The matrix pool composition was based on viral gene order and not based on absolute overall ranking (color code in **Figure 1B**). This matrix approach enabled screening of 50 H2-Db and 50 H2-Kb predicted epitopes for T cell activation upon VSV-GP immunization of C57BL/6J mice.

### 3.2 Peptide pool screening of H2-Db and H2-Kb presented epitopes

**Figure 2:**
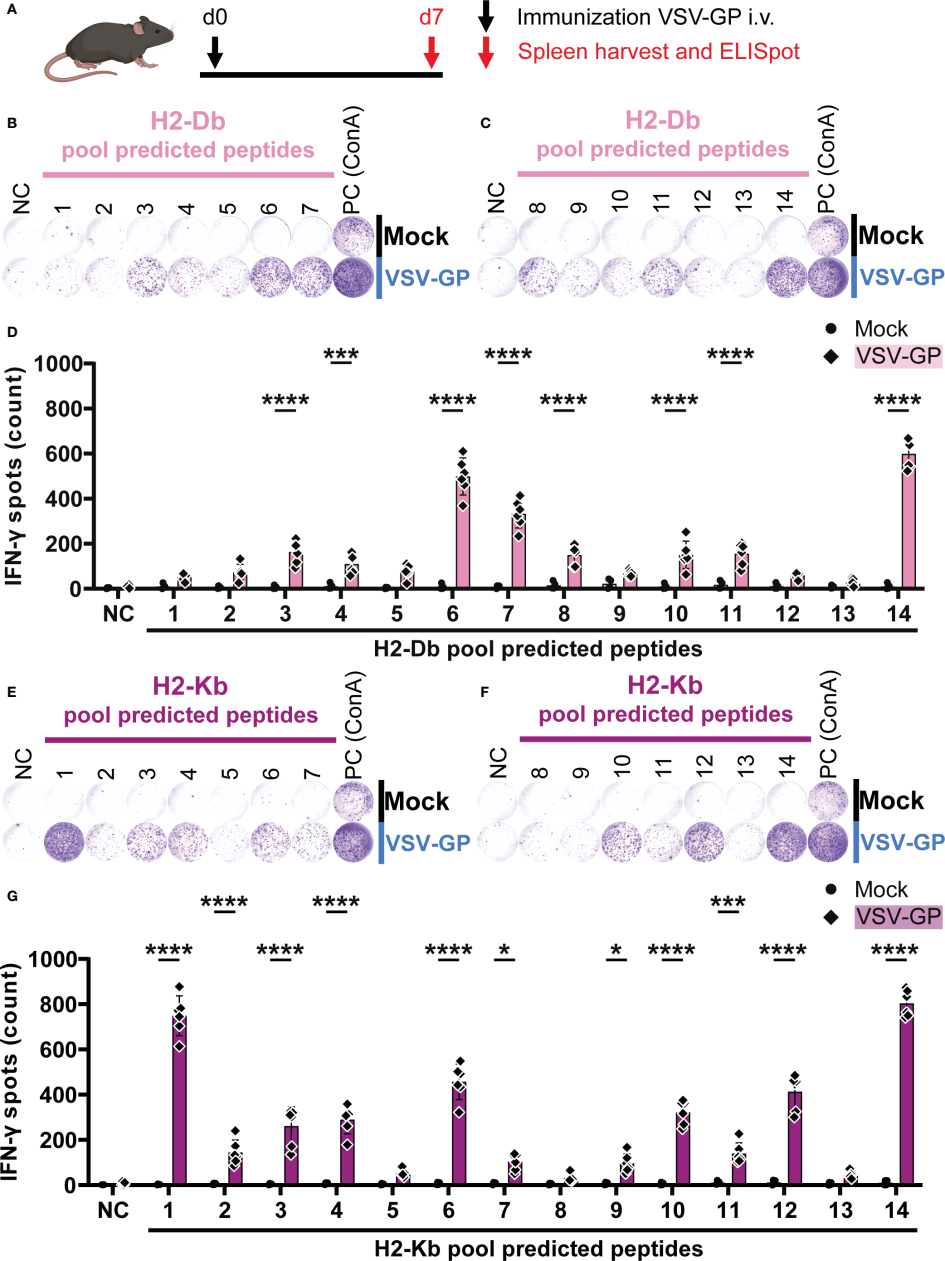
H2-Db and H2-Kb peptide pool screening using IFN-γ ELISpot identifies activating peptide pools. **(A)** Schematic representation of the experimental setup. C57BL/6J mice were intravenously (i.v.) immunized with VSV-GP on day 0 (d0). On day 7 (d7), spleens were harvested for IFN-γ ELISpot. **(B,C,E,F)** Representative images of IFN-γ ELISpots using 2.5 x 10^5^ splenocytes per well upon stimulation with 35 µg/ml H2-Db peptide pools 1-7 **(B)** and 8-14 **(C)**, and with 35 µg/ml H2-Kb peptide pools 1-7 **(E)** and 8-14 **(F)**. The negative control (NC) is unstimulated cells and the positive control (PC) is 5 µg/ml ConA. **(D,G)** Quantification of IFN-γ ELISpots with H2-Db peptide pools **(D)** and H2-Kb peptide pools **(G)** (n=4 for mock, n=6 for VSV-GP, from two independently performed experiments). Significant differences between mock and VSV-GP treatment are indicated with asterisks (tested using two-way ANOVA with Sidak’s multiple comparison). *p < 0.05; ***p ≤ 0.001; ****p ≤ 0.0001.

In order to assess T cell activation by the peptide pools, C57BL/6J mice were immunized intravenously with 10^8^ TCID_50_ VSV-GP. One week post immunization, splenocytes were harvested and stimulated with the peptide pools in an IFN-γ ELISpot (**Figure 2A**). During stimulation, the peptides are hypothetically taken up by antigen-presenting cells (APCs), and presented on mouse MHC-I alleles H2-Db and H2-Kb. If the peptides are presented this may lead to activation of anti-viral CD8^+^ T cells with TCRs that recognize the H2-Db-epitope or H2-Kb-epitope complexes, leading to IFN-γ release, as shown in exemplary ELISpots for H2-Db (**Figures 2B, C**) and H2-Kb (**Figures 2E, F** **).** A significant difference in the number of IFN-γ spots by splenocytes of VSV-GP immunized mice compared to splenocytes of mock mice was detected for eight H2-Db peptide pools (**Figure 2D**) and eleven H2-Kb peptide pools (**Figure 2G**). Based on this group analysis, matrix deconvolution was performed by crossing out non-significant peptide pools to select the individual H2-Db and H2-Kb candidate epitopes to be tested individually (**Supplementary Figure 3**).

### 3.3 Identification of H2-Db and H2-Kb presented VSV-GP T cell epitopes

In order to test the individual epitope candidates, a similar experimental setup was used as for the peptide pools. Based on the matrix deconvolution, 17 H2-Db and 31 H2-Kb peptides were selected as candidates. These individual candidates were assessed with regard to the respective vertical peptide pool to determine significant IFN-γ secretion following T cell activation upon stimulation with epitope candidates.

**Figure 3:**
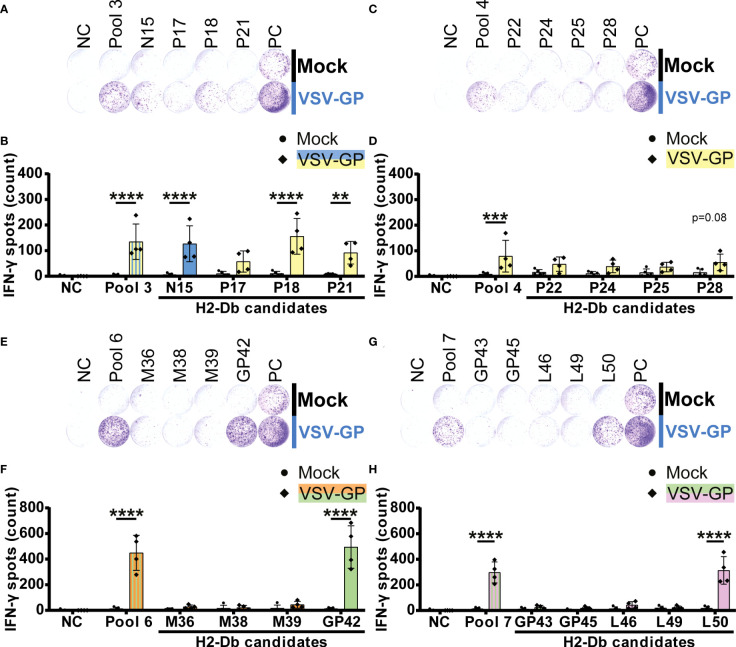
H2-Db individual peptide testing using IFN-γ ELISpot identifies H2-Db presented VSV-GP T cell epitopes. **(A,C,E,G)** Representative images of IFN-γ ELISpots using 2.5 x 10^5^ splenocytes per well upon stimulation with 35 µg/ml peptide pool or 5 µg/ml individual peptide. The negative control (NC) is unstimulated cells, and the positive control (PC) is 5 µg/ml ConA. **(B,D,F,H)** Quantification of IFN-γ ELISpots (n=5 for mock, n=4 for VSV-GP, from two independently performed experiments). Significant differences between mock and VSV-GP treatment are indicated with asterisks (tested using two-way ANOVA with Sidak’s multiple comparison). Bar color represents from which viral protein the peptide or peptide pool is derived. **p ≤ 0.01; ***p ≤ 0.001; ****p ≤ 0.0001.

In total, five H2-Db peptides significantly induced IFN-γ spot formation by splenocytes of VSV-GP immunized mice (**Figure 3**). Of those, one VSV-N epitope (N15) and two VSV-P epitopes (P18 and P21) were present in H2-Db pool 3 (**Figures 3A, B**). Additionally, we identified one LCMV-GP epitope (GP42) in H2-Db pool 6 (**Figures 3E, F**) and one VSV-L epitope (L50) in H2-Db pool 7 (**Figures 3G, H**). No significant epitopes were detected in H2-Db pool 4 (**Figures 3C, D**). The magnitude of the induced IFN-γ secretion differed per epitope, where GP42 induced the highest T cell activation based on the IFN-γ spot count.

**Figure 4:**
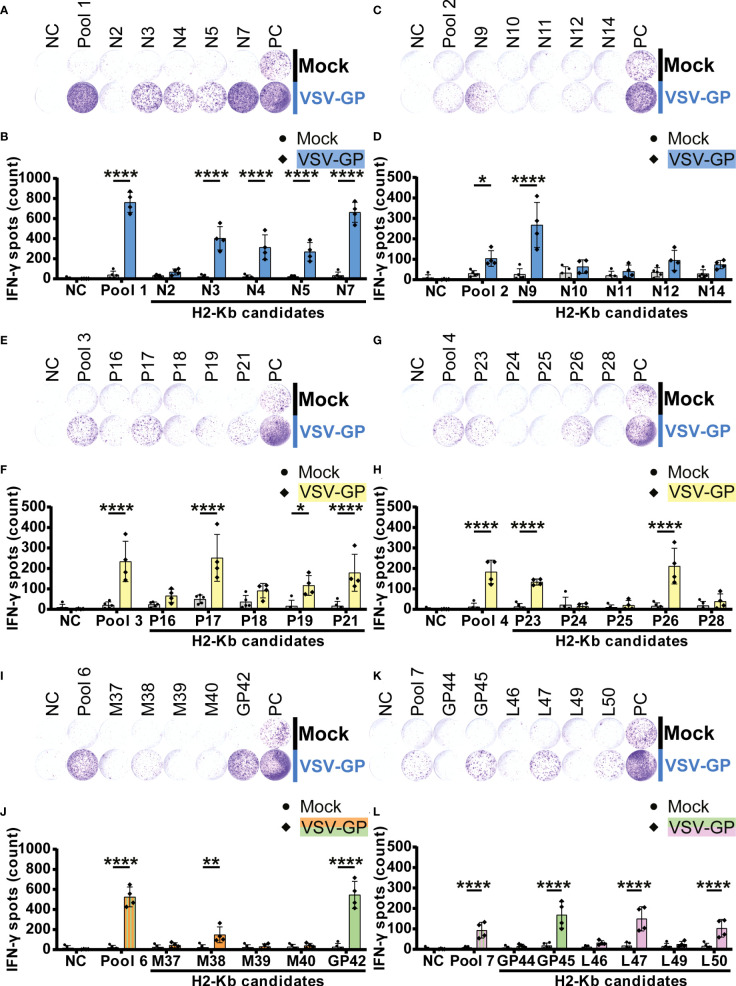
H2-Kb individual peptide testing using IFN-γ ELISpot identifies H2-Kb presented VSV-GP T cell epitopes.**(A,C,E,G,I,K)** Representative images of IFN-γ ELISpots using 2.5 x 10^5^ splenocytes per well upon stimulation with 35 µg/ml peptide pool or 5 µg/ml individual peptide. The negative control (NC) is unstimulated cells, and the positive control (PC) is 5 µg/ml ConA. **(B,D,F,H,J,L)** Quantification of IFN-γ ELISpots (n=5 for mock, n=4 for VSV-GP, from two independently performed experiments). Significant differences between mock and VSV-GP treatment are indicated with asterisks (tested using two-way ANOVA with Sidak’s multiple comparison). Bar color represents from which viral protein the peptide or peptide pool is derived. *p < 0.05; **p ≤ 0.01; ****p ≤ 0.0001.

Significant IFN-γ secretion by splenocytes of VSV-GP immunized mice was induced by fifteen H2-Kb peptides **(** **Figure 4** **).** We detected five significant H2-Kb epitopes of VSV-N (N3, N4, N5, N7 and N9; **Figures 4A–D**) and five significant H2-Kb epitopes of VSV-P (P17, P19, P21, P23 and P26; **Figures 4E–H**). Furthermore, one VSV-M epitope (M38; **Figures 4I, J**), two LCMV-GP epitopes (GP42 and GP45; **Figures 4I–L**) and two VSV-L epitopes (L47 and L50; **Figures 4K, L**) significantly caused IFN-γ spot formation. Similarly to H2-Db epitopes, the number of IFN-γ spots differed between different H2-Kb epitopes, of which epitope N7 showed the highest IFN-γ spot count.

Interestingly, even though the number of T cell epitopes presented by H2-Db was lower than by H2-Kb, the average IFN-γ spot count induced by H2-Db presented VSV-GP epitopes was similar compared to the average number of IFN-γ spots induced by H2-Kb presented epitopes **(** **Supplementary Figure 4**). The maximal number of IFN-γ spots was induced by H2-Kb-N7. Altogether, these results have identified 20 VSV-GP T cell epitopes presented by MHC-I alleles H2-Db and H2-Kb in C57BL/6J mice.

### 3.4 ICS validation of H2-Db and H2-Kb presented VSV-GP T cell epitopes

**Figure 5:**
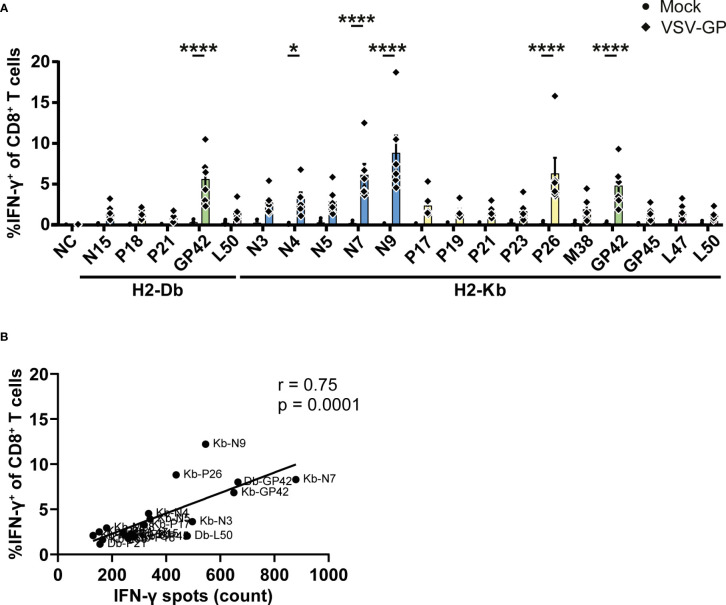
Intracellular cytokine staining upon stimulation with the identified VSV-GP T cell epitopes. **(A)** Frequencies of IFN-γ^+^ CD8^+^ T cells of splenocytes from mock or VSV-GP treated mice upon stimulation with individual peptides (10 µg/ml). The negative control (NC) is unstimulated cells (n=6 for mock, n=6 for VSV-GP, from three independently performed experiments). Significant differences between mock and VSV-GP treatment are indicated with asterisks (tested using two-way ANOVA with Sidak’s multiple comparison). *p < 0.05; ****p ≤ 0.0001. **(B)** Correlation of matching ICS and IFN-γ ELISpots (using Pearson correlation coefficient). Bar color represents from which viral protein the epitope is derived.

In order to further validate our results, intracellularly produced IFN-γ was stained in CD8^+^ T cells upon stimulation with the identified epitopes. The gating strategy is shown in **Supplementary Figure 5A**. In total, one H2-Db peptide and five H2-Kb epitopes induced significant IFN-γ^+^ CD8^+^ T cells by splenocytes of VSV-GP immunized mice (**Figure 5A**). Of note, none of the identified epitopes induced IFN-γ^+^ CD4^+^ T cells (**Supplementary Figure 5B**). The magnitude of the response differed per epitope but correlated significantly with the IFN-γ spot count of the corresponding ELISpot assays (**Figure 5B**; **Supplementary Figure 5C**).

### 3.5 Characteristics of H2-Db and H2-Kb presented VSV-GP T cell epitopes

**Figure 6:**
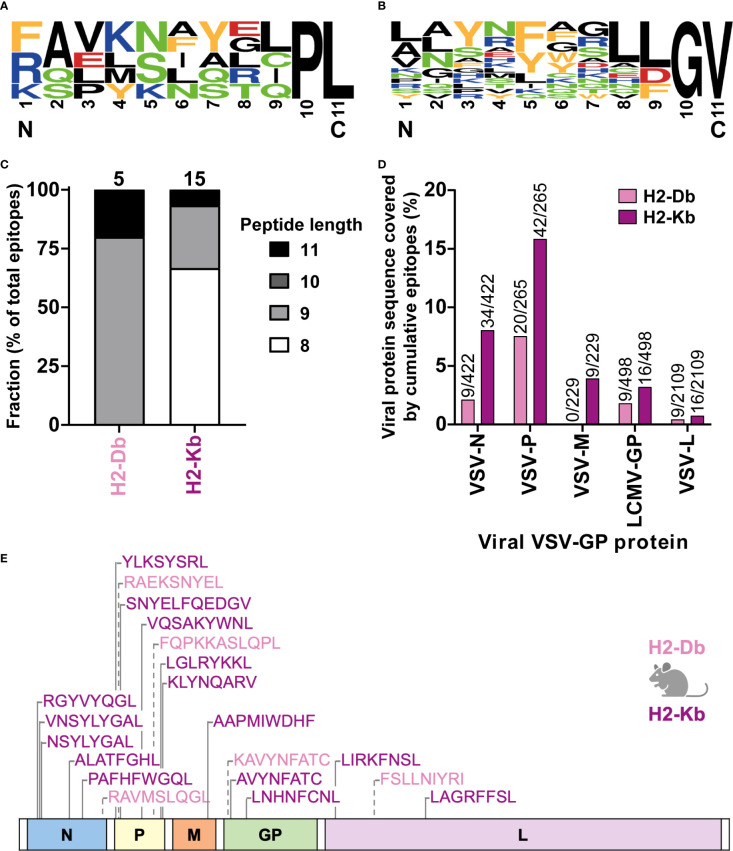
H2-Db and H2-Kb presented VSV-GP T cell epitopes differ in peptide sequence logos, peptide length, and viral protein immunogenicity but not in distribution across VSV-GP proteome. **(A, B)** Sequence logos of five H2-Db presented VSV-GP epitopes **(A)** and fifteen H2-Kb presented VSV-GP epitopes **(B)**. Letter size indicates the frequency of the AA at that position. Positively charged AAs are indicated in blue, negatively charged AAs in red, aromatic AAs in yellow, polar AAs in green, and non-polar AAs in black. **(C)** Relative distribution of peptide lengths plotted per MHC-I allele for all identified VSV-GP T cell epitopes. Numbers above bars represent total identified epitopes per MHC-I allele. **(D)** Viral protein sequence covered by the identified epitopes. Numbers above bars represent summed AAs covered by identified epitopes divided by the viral protein AA length. **(E)** Schematic overview of the VSV-GP T cell epitopes in the VSV-GP proteome. The epitope sequences have been indicated per MHC-I allele tested (H2-Db in pink, H2-Kb in purple) and their position in the VSV-GP proteome.

In total, we identified five H2-Db presented VSV-GP T cell epitopes (**Supplementary Table 1**) and fifteen H2-Kb presented VSV-GP T cell epitopes (**Supplementary Table 2**). H2-Db presented epitopes showed a sequence motif containing mostly polar residues in the middle of the peptide, mainly at position 5, and a non-polar C-terminus of the peptide (**Figure 6A**). H2-Kb presented epitopes, on the other hand, showed mostly aromatic residues at positions 3 and 5 and, similarly to H2-Db presented epitopes, a non-polar C-terminus of the peptide (**Figure 6B**). Generally, H2-Kb presented epitopes were shorter compared to H2-Db presented epitopes (**Figure 6C**). Looking at the viral proteome presented by the different MHC-I alleles, epitopes were identified from all five VSV-GP proteins, where H2-Kb presented epitopes of each viral protein, whereas no VSV-M epitopes were identified for H2-Db (**Figure 6D**). The identified epitopes were scattered throughout the VSV-GP proteome and similarly distributed for both MHC-I alleles (**Figure 6E**).

## 4 Discussion

Oncolytic viruses, such as VSV-GP, present as a promising therapeutic modality for cancer. In recent years the focus of oncolytic virotherapy has shifted increasingly toward its potential to induce anti-tumor immune responses. However, application of such therapeutic viruses still triggers a substantial anti-viral T cell activity, the implication of which is subject of ongoing debate (10, 11). On one hand, anti-viral CD8^+^ T cells may interfere with the induction of potential anti-tumor CD8^+^ T cells due to the often immunodominant nature of viral epitopes compared to tumor-associated or tumor-specific antigens (8). On the other hand, through indirect mechanisms, such as the general immune-promoting conversion of the tumor microenvironment, virus induced immune activation may also facilitate enhanced activity of anti-tumor T cells (11). Of note, whereas monitoring of T cell responses against tumor-related antigens has long been a mainstay in preclinical studies, the qualitative and quantitative characterization of viral antigen-specific T cell levels have received much less attention. Therefore, in this study, we identified the VSV-GP T cell epitopes presented by H2-Db and H2-Kb in the mouse model widely used for OV research, C57BL/6J mice.

The identified epitopes presented by the abovementioned MHC-I alleles show characteristics that are in accordance with literature. We identified four nonamers and one undecamer as H2-Db presented VSV-GP epitopes. H2-Db mostly presents nonamers (25, 26) and can present longer peptides, such as undecamers, but rarely shorter peptides, such as octamers, due to the ridge in the cleft of the H2-Db molecule (27). One of the pockets in the center of the peptide binding groove of H2-Db, the C pocket, is important for binding of the peptide (26). This C pocket interacts with the AA at position 5, which is therefore called an anchor residue (26, 27). Asparagine (N) is usually found at position 5 of peptides binding to H2-Db (25, 26), which we also mostly observed in our identified H2-Db presented epitopes. Another anchor residue is position 9 of the peptide, or position 11 for undecamers (26). We mainly found the hydrophobic residue leucine (L), which has been described previously (26, 27). Lastly, alanine (A), glutamine (Q), and serine (S) were frequently present at the anchor residue in position 2, which, again, is in line with literature (25, 26, 28, 29).

H2-Kb presents shorter peptides compared to H2-Db. H2-Kb binds mostly octamers and, to a lesser extent, nonamers (25, 26), due to the structure of the C-terminal end of the peptide binding groove of the H2-Kb molecule (30), fitting our data on H2-Kb presented VSV-GP T cell epitopes. Additionally, we almost exclusively identified H2-Kb presented peptides with an aromatic residue at position 5; phenylalanine (F) or tyrosine (Y). These are known AAs for this anchor residue (25, 26, 31), since H2-Kb allows binding of bulkier residues at position 5, such as aromatic AAs, due to the structure of its C pocket, which is deeper compared to the C pocket of H2-Db (25, 26). The other anchor residue at position 8, or at the last position for longer peptides, usually contains a leucine (L) or, less frequently, a valine (V) (25, 26, 31), again fitting our observations.

Only some of the predicted candidate peptides induced IFN-γ spot formation. This could be due to multiple causes. Firstly, the bioinformatic prediction algorithms may have falsely predicted binding of these viral peptides to the indicated MHC-I molecule. Even though the epitope prediction tools used in this study are among the top-performing algorithms, all bioinformatic tools have a false discovery rate (32). On the other hand, NetMHCpan 4.0 and MHCflurry have a good performance in benchmark analyses of T cell epitopes in vaccinia virus (VACV) infected C57BL/6 mice (33). Hence, current versions of these approaches, NetMHCpan 4.1 (17) and MHCflurry 2.0 (18), were used for prediction of viral epitopes in this study. Although in recent years data for neoantigens bound to MHC class I molecules have become available by mass spectrometry analysis and have been used to train algorithms, predictions still rely on the immune epitope database (IEDB), which contains many different other sources including bacterial or viral epitopes. For example, the IEDB currently lists more than 4,800 epitopes of viral origin that have been shown to be ligands for MHC class I molecules or are T cell epitopes in mice. Accordingly, training data for the netMHCpan 4.1 binding affinity approach (BA) include data from Alvarez et al. (34) and IEDB (35) with >50,000 epitopes binding to MHC-I (<500 nM) and cover 170 MHC alleles, including H2-Db and H2-Kb. Additionally, we combined several epitope prediction tools to increase the likelihood of selecting true epitopes.

Secondly, an MHC-peptide complex does not necessarily induce TCR recognition. The conformation of the presented peptide could be optimal for MHC binding, but suboptimal for TCR recognition (36). Our approach does not distinguish between MHC binding and TCR recognition, since our read-out of IFN-γ secretion is a consequence of the occurrence of both processes. Other approaches to identify T cell epitopes, such as MHC binding assays (37) or MHC ligand purification and subsequent mass spectrometry (38), can detect MHC binding of viral epitopes, but cannot determine whether the identified epitopes induce a T cell response. Therefore, we used a bioinformatic guided approach using several epitope prediction tools and subsequent ELISpot assays to identify the VSV-GP T cell epitopes that induce a T cell response upon VSV-GP immunization of C57BL/6J mice.

Thirdly, T cells directed against the MHC-peptide complex may have been negatively selected in the thymus (39) when these T cells have high avidity for the MHC-self peptide complex (40), explaining the absence of T cells recognizing such complexes. However, in our study, we included a filter for dissimilarity against the mouse proteome and therefore none of the identified epitopes are homologous to the mouse proteome.

Lastly, *in vivo* presented epitopes might differ from the epitopes we used since *in vivo* epitope processing might not generate these predicted epitopes. *In vivo* processing of the viral proteins by the antigen presentation machinery affects which epitopes are presented by MHC-I and consequently inducing T cell responses (41). Several parts of the MHC-I antigen presentation pathway could affect which epitopes are generated and presented *in vivo*, such as degradation by the proteasome and cytosolic peptidases, as well as TAP transport (13). Nonetheless, antigen processing was taken into account by the MHCflurry prediction tool used in this study (18), and this algorithm predicted most candidates as strong or weak, suggesting these epitopes will likely be processed *in vivo* as well.

We further validated our identified epitopes using ICS. Although not all epitopes were significantly inducing IFN-γ^+^ CD8^+^ T cells, distinct positive populations of IFN-γ^+^ CD8^+^ T cells were observed for all tested peptides while none of the epitopes induced IFN-γ^+^ CD4^+^ T cells. Additionally, this does not indicate these epitopes are falsely identified, but merely indicates the lack of sensitivity that is associated with ICS compared to ELISpot (42). This is emphasized by the strong, significant correlation of our ICS and ELISpot results.

In our study we have also identified four epitopes presented by H2-Db and H2-Kb that have previously been described in literature, indicating our unbiased approach also, rightfully, predicts known immunogenic epitopes.

H2-Kb-N7 (RGYVYQGL) is the only H2-Kb presented VSV-N epitope currently described. H2-Kb-N7 is positioned in VSV-N at amino acids 52-59 and induces both T cell activation and cytotoxicity (43, 44). Additionally, a tetramer with this epitope is currently used as the main read-out of anti-VSV-GP T cell responses in VSV-GP research (7, 8). One other VSV-N epitope has been described (MPYLIDFGL; position 275-283), but is presented by MHC-I allele H2-Ld of BALB/c mice (45).

The second, third, and fourth known epitopes are derived from the glycoprotein GP of LCMV, for which the natural host is the house mouse, and therefore more widely studied in the context of mouse anti-viral immune responses. H2-Kb-GP45 (LNHNFCNL), located at amino acids 118-125 in LCMV-GP, induces IFN-γ secretion by CD8^+^ T cells by flow cytometry (46, 47), similarly to our observations. Furthermore, H2-Kb-GP42 (AVYNFATC) is located at amino acids 34-41 of LCMV-GP, and it induces cytotoxicity (48) and IFN-γ secretion (49, 50) by CD8^+^ T cells. The last previously described epitope is H2-Db-GP42 (KAVYNFATC). This is an overlapping epitope with the third known epitope but presented by H2-Db, and not by H2-Kb (49) and induces IFN-γ secretion (51).

At the time of this study, no VSV-P, VSV-M or VSV-L epitopes had been described in literature. Therefore, this is the first study showing, in some cases very high, T cell activation upon stimulation with three novel H2-Db and eight novel H2-Kb VSV-GP epitopes derived from VSV-P, VSV-M and VSV-L, in addition to one novel H2-Db VSV-N epitope and four novel H2-Kb VSV-N epitopes.

In general, the immunogenicity of the five VSV-GP proteins differed based on the AA sequence of the proteins covered and the number of epitopes identified. VSV-P seemed the most immunogenic, as in total most identified epitopes originate from this viral protein and the highest percentage of the viral protein sequence was cumulatively covered by the identified epitopes of both MHC-I alleles. After VSV-P, the immunogenicity of VSV-N was the highest, and then VSV-M, LCMV-GP, and lastly VSV-L. A decrease in immunogenicity of lower expressed viral proteins is in line with observations of other viruses with a similar, negative-strand RNA genome, such as Ebola virus and measles virus. In Ebola survivors, CD8^+^ T cell responses decrease against lower expressed proteins (52), suggesting either fewer epitopes are present or less strong immune responses are induced against those epitopes. Additionally, the number of identified epitopes is comparable to the measles virus (53).

Moreover, the identification of VSV-GP T cell epitopes could also have some bearing on the application of the VSV Ebola vaccine (VSV-EBOV). In this replication-competent VSV-based vaccine, the VSV glycoprotein has been replaced with the Ebola virus glycoprotein (EBOV) (54), and this vaccine has been FDA approved (55). Application of the vaccine could induce T cell mediated viral vector immunity, and repeated application could boost this vector immunity and limit vaccine efficacy. Therefore, the identification of VSV-GP epitopes in preclinical mouse models could also be relevant for development and (repeated) application of VSV-based vaccines against numerous pathogens (56) to study T cell immunity in more detail.

In conclusion, this study showed that mouse MHC-I alleles H2-Db and H2-Kb present VSV-GP epitopes which induce T cell activation. Four novel H2-Db presented and twelve novel H2-Kb presented VSV-GP T cell epitopes were identified, including epitopes of VSV-P, VSV-M and VSV-L for which – to our knowledge – no epitopes were previously described. These findings may support the assessment of anti-viral immunity against novel VSV-GP variants in preclinical development. Lastly, these findings could aid in further development of novel VSV-based vaccines and therapeutics.

## Data availability statement

The original contributions presented in the study are included in the article/**Supplementary Material**. Further inquiries can be directed to the corresponding author.

## Ethics statement

The animal study was reviewed and approved by the Institutional Review Board of the Medical University of Innsbruck (ZVTA) and the Federal Ministry of the Republic of Austria for Education, Science and Research (BMBWF; license 2020-0.475.503).

## Author contributions

GW and KD conceptualized the study. SV, SD, LP, KD, and GW were involved in the design of the experiments. RG, GF, SV, and HH performed bioinformatic analyses. SV, SD, and LP carried out the experiments. SV, SD, and LP performed analysis and interpretation of data, and wrote the manuscript. GW, KD, and HH revised the manuscript. All authors contributed to the article and approved the submitted version.
