# Supplementary material for: Prediction and validation of murine MHC class I epitopes of the recombinant virus VSV-GP
Source: Front Immunol. 2023 Jan 19;13:1100730. doi: 10.3389/fimmu.2022.1100730 (PMC9893851; doi:10.3389/fimmu.2022.1100730)
Supplement: Supplementary file 1 [file DataSheet_1.pdf]

Supplementary Material

1 Supplementary Figures and Tables

1.1 Supplementary Figures

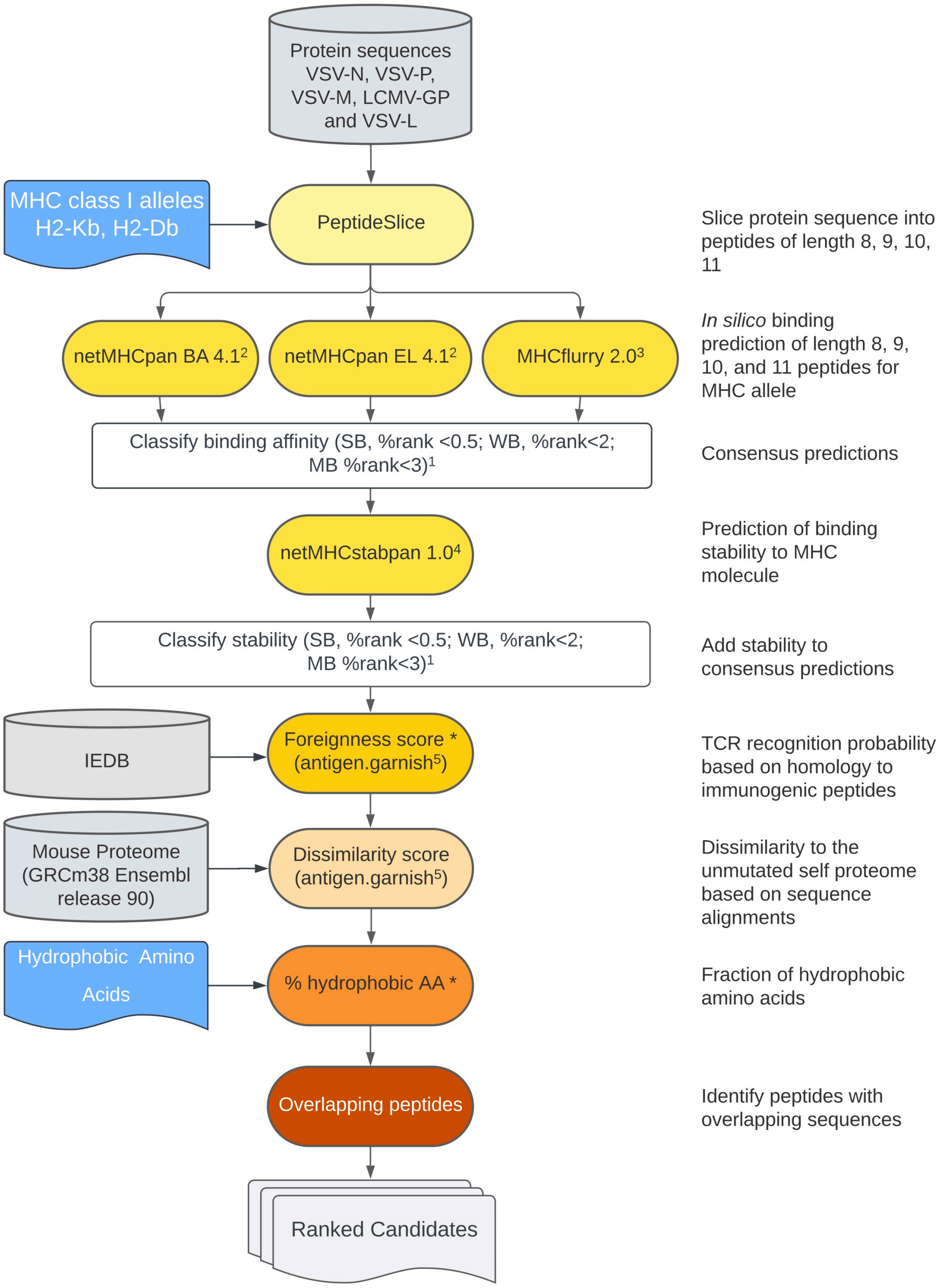

Supplementary Figure 1: Workflow for ranking MHC class I epitopes from viral proteins. The amino acid sequences of the viral proteins VSV-N, VSV-P, VSV-M, LCMV-GP and VSV-L were cut into peptides of length 8-11 using a sliding window approach. Epitope binding predictions were performed using the algorithms netMHCpan 4.1 (based on different data basis/method for binding affinity (BA) and eluted ligands (EL)) as well as MHCflurry 2.0 for the MHC alleles of interest. Predicted MHC class I binding epitopes were ranked by the consensus results of the three methods, which categorized them as strong binders (SB, %rank < 0.5), weak binders (WB, %rank < 2) or marginal binders (MB, %rank < 3), respectively. Peptides with similar ranking were further prioritized by binding stability of the peptide-MHC complex predicted with netMHCstabpan and dissimilarity to the unmutated mouse proteome by sequence alignments using the R package antigen.garnish. If applicable, the final candidate lists for each viral protein and each MHC class I allele were limited to a maximum of three peptides with an overlapping sequence of more than six amino acids.

[1] Strong binder (SB); weak binder (WB); marginal binder (MB).

[2] Reynisson B, Alvarez B, Paul S, Peters B, Nielsen M. NetMHCpan-4.1 and NetMHCIIpan-4.0: Improved predictions of MHC antigen presentation by concurrent motif deconvolution and integration of MS MHC eluted ligand data. Nucleic Acids Res (2020) 48:W449–W454.

[3] O'Donnell TJ, Rubinsteyn A, Laserson U. MHCflurry 2.0: Improved Pan-Allele Prediction of MHC Class I-Presented Peptides by Incorporating Antigen Processing. Cell Syst (2020) 11:42–48.

[4] Rasmussen M, Fenoy E, Harndahl M, Kristensen AB, Nielsen IK, Nielsen M, Buus S. Pan-Specific Prediction of Peptide–MHC Class I Complex Stability, a Correlate of T Cell Immunogenicity. J Immunol (2016) 197:1517–1524.

[5] Richman LP, Vonderheide RH, Rech AJ. Neoantigen Dissimilarity to the Self-Proteome Predicts Immunogenicity and Response to Immune Checkpoint Blockade. Cell Syst (2019) 9:375-382.e4.

[*] The percentage of hydrophobic amino acids and the foreignness score were computed but were not used for epitope ranking.

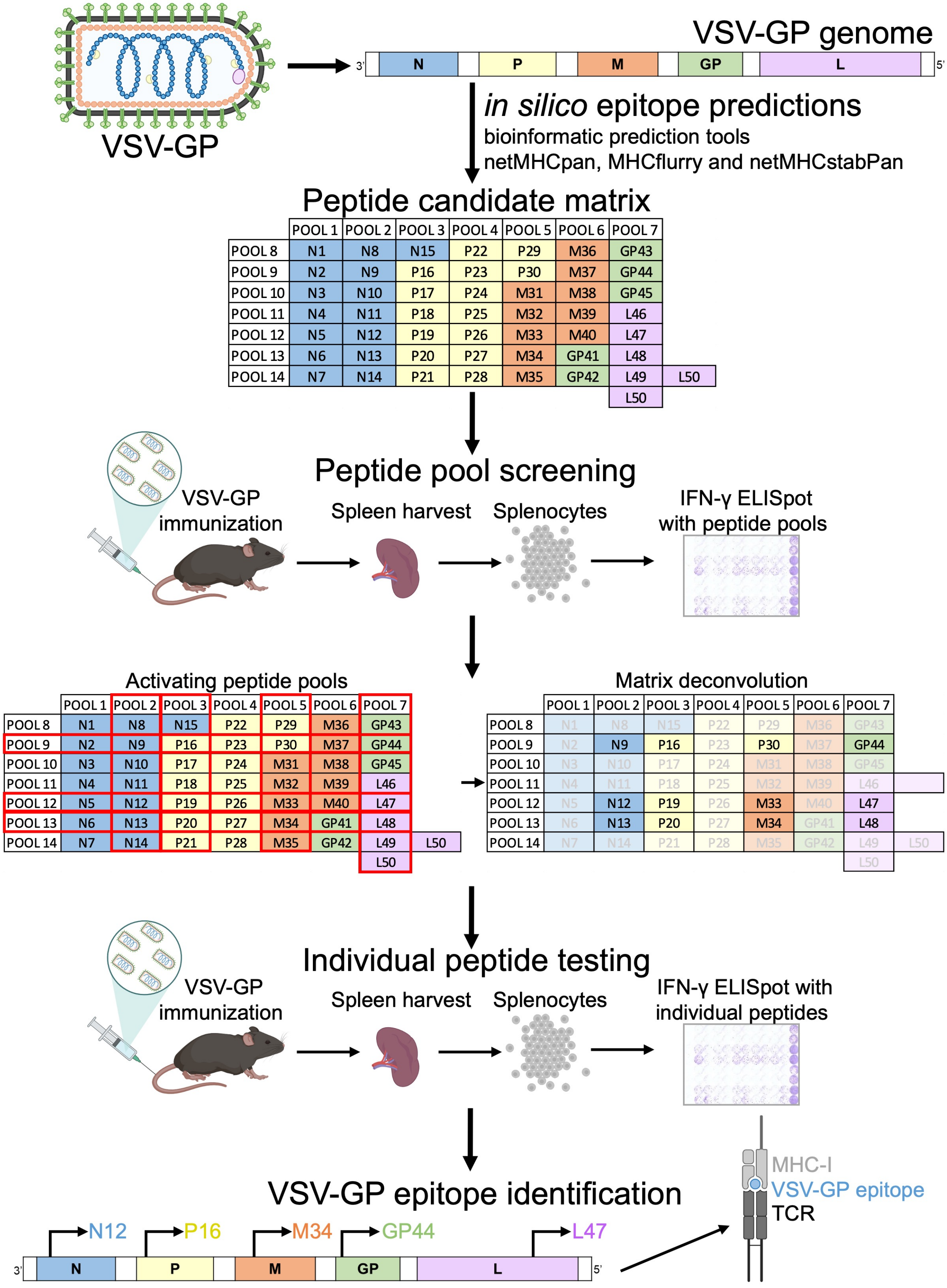
VSV-GP
VSV-GP genome
3'
N
P
M
GP
L
5'
in silico epitope predictions
bioinformatic prediction tools
netMHCpan, MHCflurry and netMHCstabPan
Peptide candidate matrix
POOL 1 POOL 2 POOL 3 POOL 4 POOL 5 POOL 6 POOL 7
POOL 8 N1 N8 N15 P22 P29 M36 GP43
POOL 9 N2 N9 P16 P23 P30 M37 GP44
POOL 10 N3 N10 P17 P24 M31 M38 GP45
POOL 11 N4 N11 P18 P25 M32 M39 L46
POOL 12 N5 N12 P19 P26 M33 M40 L47
POOL 13 N6 N13 P20 P27 M34 GP41 L48
POOL 14 N7 N14 P21 P28 M35 GP42 L49 L50
L50
Peptide pool screening
VSV-GP immunization
Spleen harvest
Splenocytes
IFN-γ ELISpot with peptide pools
Activating peptide pools
Matrix deconvolution
N9 P16 P30 GP44
N12 P19 M33 L47
N13 P20 M34 L48
Individual peptide testing
VSV-GP immunization
Spleen harvest
Splenocytes
IFN-γ ELISpot with individual peptides
VSV-GP epitope identification
N12
P16
M34
GP44
L47
3'
N
P
M
GP
L
5'
MHC-I
VSV-GP epitope
TCR

Supplementary Figure 2: Overview of methodology. Based on the amino acid (AA) sequences of the viral proteins in silico epitope predictions were performed. Due to the decreasing viral protein expression, a decreasing number of candidate epitopes were included in the peptide candidate matrix. In this exemplary matrix, the same predicted epitope was included in both a horizontal and a vertical pool (e.g. N3 was included in pools 1 and 10). The peptides of the predicted epitopes were synthesized and subsequently the peptide pools were screened using splenocytes of VSV-GP immunized mice in an IFN-γ ELISpot. The significantly activating peptide pools were used to perform matrix deconvolution, for the selection of the individual peptide candidates. These were tested using splenocytes of VSV-GP immunized mice in an IFN-γ ELISpot, to identify the VSV-GP T cell epitopes that are presented by MHC-I and recognized by anti-viral T cells with their TCR.

A

	POOL 1	POOL 2	POOL 3	POOL 4	POOL 5	POOL 6	POOL 7	
POOL 8	H2-Db-N1	H2-Db-N8	H2-Db-N15	H2-Db-P22	H2-Db-P29	H2-Db-M36	H2-Db-GP43	
POOL 9	H2-Db-N2	H2-Db-N9	H2-Db-P16	H2-Db-P23	H2-Db-P30	H2-Db-M37	H2-Db-GP44	
POOL 10	H2-Db-N3	H2-Db-N10	H2-Db-P17	H2-Db-P24	H2-Db-M31	H2-Db-M38	H2-Db-GP45	
POOL 11	H2-Db-N4	H2-Db-N11	H2-Db-P18	H2-Db-P25	H2-Db-M32	H2-Db-M39	H2-Db-L46	
POOL 12	H2-Db-N5	H2-Db-N12	H2-Db-P19	H2-Db-P26	H2-Db-M33	H2-Db-M40	H2-Db-L47	
POOL 13	H2-Db-N6	H2-Db-N13	H2-Db-P20	H2-Db-P27	H2-Db-M34	H2-Db-GP41	H2-Db-L48	
POOL 14	H2-Db-N7	H2-Db-N14	H2-Db-P21	H2-Db-P28	H2-Db-M35	H2-Db-GP42	H2-Db-L49	H2-Db-L50
							H2-Db-L50	

B

	POOL 1	POOL 2	POOL 3	POOL 4	POOL 5	POOL 6	POOL 7	
POOL 8	H2-Kb-N1	H2-Kb-N8	H2-Kb-N15	H2-Kb-P22	H2-Kb-P29	H2-Kb-M36	H2-Kb-GP43	
POOL 9	H2-Kb-N2	H2-Kb-N9	H2-Kb-P16	H2-Kb-P23	H2-Kb-P30	H2-Kb-M37	H2-Kb-GP44	
POOL 10	H2-Kb-N3	H2-Kb-N10	H2-Kb-P17	H2-Kb-P24	H2-Kb-M31	H2-Kb-M38	H2-Kb-GP45	
POOL 11	H2-Kb-N4	H2-Kb-N11	H2-Kb-P18	H2-Kb-P25	H2-Kb-M32	H2-Kb-M39	H2-Kb-L46	
POOL 12	H2-Kb-N5	H2-Kb-N12	H2-Kb-P19	H2-Kb-P26	H2-Kb-M33	H2-Kb-M40	H2-Kb-L47	
POOL 13	H2-Kb-N6	H2-Kb-N13	H2-Kb-P20	H2-Kb-P27	H2-Kb-M34	H2-Kb-GP41	H2-Kb-L48	
POOL 14	H2-Kb-N7	H2-Kb-N14	H2-Kb-P21	H2-Kb-P28	H2-Kb-M35	H2-Kb-GP42	H2-Kb-L49	H2-Kb-L50
							H2-Kb-L50	

Supplementary Figure 3: H2-Db and H2-Kb matrix deconvolution to identify individual peptide candidates. A-B) Based on the peptide pool screening, non-significant peptide pools were crossed out (indicated by grey text and fainter colors), thereby identifying the individual H2-Db peptide candidates (A) and individual H2-Kb peptide candidates (B).

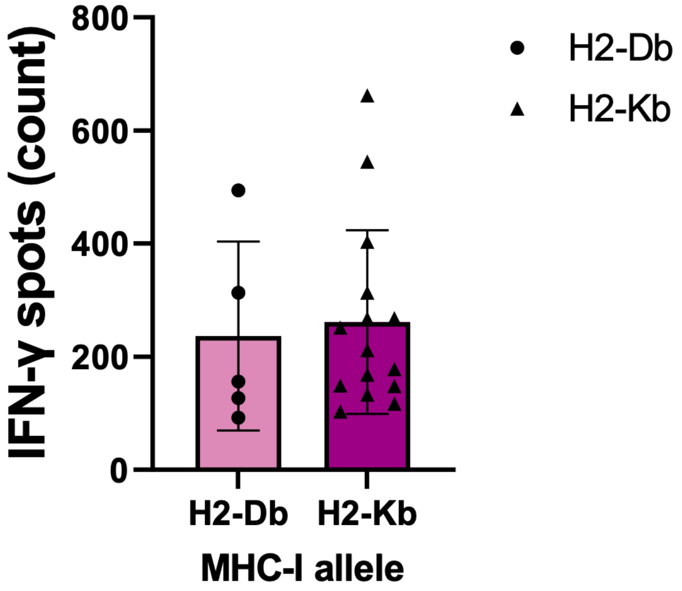

Supplementary Figure 4: VSV-GP T cell epitopes presented by MHC-I alleles H2-Db and H2-Kb. Average number of IFN-γ spots of each VSV-GP T cell epitope per MHC-I allele. No statistically significant difference in mean number of IFN-γ spots was detected between H2-Db and H2-Kb (Mann□Whitney U test; p=0.62).

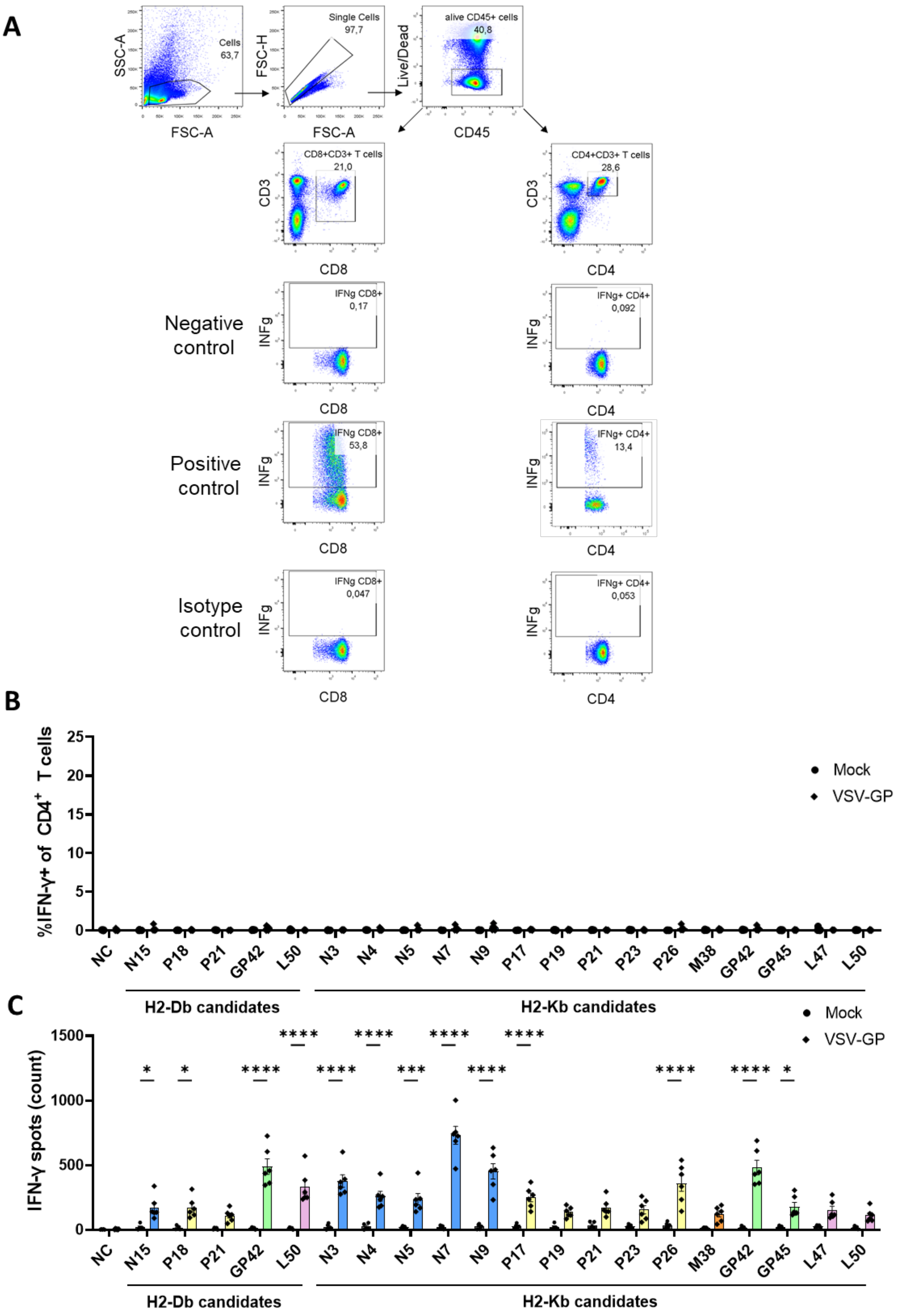
A
SSC-A
FSC-A
Cells
63,7
FSC-H
Single Cells
97,7
Live/Dead
CD45
alive CD45+ cells
40,8
CD3
CD8
CD8+CD3+ T cells
21,0
CD4
CD4+CD3+ T cells
28,6
Negative control
INFg
IFNg CD8+
0,17
IFNg+ CD4+
0,092
Positive control
IFNg CD8+
53,8
IFNg+ CD4+
13,4
Isotype control
IFNg CD8+
0,047
IFNg+ CD4+
0,053
B
%IFN-γ+ of CD4+ T cells
25
20
15
10
5
0
Mock
VSV-GP
NC
N15
P18
P21
GP42
L50
N3
N4
N5
N7
N9
P17
P19
P21
P23
P26
M38
GP42
GP45
L47
L50
H2-Db candidates
H2-Kb candidates
C
IFN-γ spots (count)
1500
1000
500
0
*
*
****
****
****
****
***
****
****
****
****
****
****
*
Mock
VSV-GP
NC
N15
P18
P21
GP42
L50
N3
N4
N5
N7
N9
P17
P19
P21
P23
P26
M38
GP42
GP45
L47
L50
H2-Db candidates
H2-Kb candidates

Supplementary Figure 5: Intracellular cytokine staining and corresponding ELISpots using VSV-GP T cell epitopes. A) gating strategy and representative plots of negative control, positive control (PHA/ionomycin) and isotype control staining. **B)** Frequencies of IFN-γ^+ $CD4^+$ T cells of splenocytes from mock or VSV-GP treated mice upon stimulation with individual peptides (10 µg/ml). **C)** Quantification of corresponding IFN-γ ELISpots, which were performed as a control for each ICS experiment (n=6 for mock, n=6 for VSV-GP, from three independently performed experiments). Significant differences between mock and VSV-GP treatment are indicated with asterisks (tested using two-way ANOVA with Sidak's multiple comparison). * $p < 0.05$; *** $p \leq 0.001$; **** $p \leq 0.0001$. Bar color represents from which viral protein the peptide is derived.

1.2 Supplementary Tables

Supplementary Table 1: VSV-GP T cell epitopes presented by H2-Db. The peptide name, average IFN-γ spot count, peptide sequence and peptide length are indicated for the significant epitopes from Figure 3.

Peptide	Average IFN-γ spot count VSV-GP treated mice	Peptide sequence	Peptide length
H2-Db-N15	127	RAVMSLQGL	9
H2-Db-P18	156	FQPKKASLQPL	11
H2-Db-P21	92	RAEKSNYEL	9
H2-Db-GP42	494	KAVYNFATC	9
H2-Db-L50	313	FSLLNIYRI	9

Supplementary Table 2: VSV-GP T cell epitopes presented by H2-Kb. The peptide name, average IFN-γ spot count, peptide sequence, peptide length and overlapping motif are indicated for the significant epitopes from Figure 4.

Peptide	Average IFN-γ spot count VSV-GP treated mice	Peptide sequence	Peptide length	Overlapping motif
H2-Kb-N3	403	NSYLYGAL	8	NSYLYGAL
H2-Kb-N4	313	VNSYLYGAL	9	NSYLYGAL
H2-Kb-N5	268	ALATFGHL	8	-
H2-Kb-N7	662	RGYVYQGL	8	-
H2-Kb-N9	267	PAFHFWGQL	9	-
H2-Kb-P17	251	LGLRYKKL	8	KL
H2-Kb-P19	117	YLKSYSRL	8	-
H2-Kb-P21	178	VQSAKYWNL	9	-
H2-Kb-P23	133	KLYNQARV	8	KL
H2-Kb-P26	211	SNYELFQEDGV	11	-
H2-Kb-M38	148	AAPMIWDHF	9	-
H2-Kb-GP42	545	AVYNFATC	8	-
H2-Kb-GP45	168	LNHNFCNL	8	-
H2-Kb-L47	149	LIRKFNSL	8	-
H2-Kb-L50	103	LAGRFFSL	8	-
